# Mesenchymal Stem and Progenitor Cells in Regeneration: Tissue Specificity and Regenerative Potential

**DOI:** 10.1155/2017/5173732

**Published:** 2017-02-13

**Authors:** Rokhsareh Rohban, Thomas Rudolf Pieber

**Affiliations:** ^1^Department of Internal Medicine, Division of Endocrinology and Diabetology, Medical University of Graz, Graz, Austria; ^2^Center for Medical Research (ZMF), Medical University of Graz, Graz, Austria

## Abstract

It has always been an ambitious goal in medicine to repair or replace morbid tissues for regaining the organ functionality. This challenge has recently gained momentum through considerable progress in understanding the biological concept of the regenerative potential of stem cells. Routine therapeutic procedures are about to shift towards the use of biological and molecular armamentarium. The potential use of embryonic stem cells and invention of induced pluripotent stem cells raised hope for clinical regenerative purposes; however, the use of these interventions for regenerative therapy showed its dark side, as many health concerns and ethical issues arose in terms of using these cells in clinical applications. In this regard, adult stem cells climbed up to the top list of regenerative tools and mesenchymal stem cells (MSC) showed promise for regenerative cell therapy with a rather limited level of risk. MSC have been successfully isolated from various human tissues and they have been shown to offer the possibility to establish novel therapeutic interventions for a variety of hard-to-noncurable diseases. There have been many elegant studies investigating the impact of MSC in regenerative medicine. This review provides compact information on the role of stem cells, in particular, MSC in regeneration.

## 1. Introduction

Being first isolated in 1966 from bone marrow, mesenchymal stem cells (MSC) are adult stromal nonhematopoietic cells, well known for their potential to differentiate into osteoblasts and osteocytes [1]. They have the ability to recruit hematopoietic host cells when forming bone in vivo [2, 3]. These cells are characterized by their spindle-like shape [4] and adherence capability to polymeric surfaces, for example, plastic. Although they are most known for their osteogenic differentiation potential, MSC have the ability to commit into all three lineages (osteogenic, chondrogenic, and adipogenic). MSC express CD105, CD73, and CD90 (cell-surface markers) but lack the expression of CD14, CD19, CD34, CD45, and HLA-DR [5]. MSC have been isolated and purified not only from bone marrow where they cooperate with hematopoietic stem cells (HSC) to form the niche, but also from various tissues, such as umbilical cord [6–9] and umbilical cord blood [10–13], white adipose tissue [14–16], placenta [17], and the amniotic membrane of placenta [4, 18–20]. The capacity of MSC to differentiate into cell lineages and develop teratoma, a preserved tumor that contains normal three-germ layer tissue and organ parts, is a reason to consider them as multipotent progenitor cells suitable for regenerative therapy.

Beside their potential to differentiate into osteoblasts in the process of osteogenesis, there have been several other regenerative roles attributed to MSC. These cells can serve as pericytes [21, 22] wrapping around blood vessels to support their structure and stability [23]. MSC have also shown the potential to integrate into the outer wall of the microvessels and arteries in many organs, such as spleen, liver, kidney, lung, pancreas, and brain [24, 25]. This led to the speculation that both bone marrow- and vascular wall-derived MSC as well as white adipose tissue-, umbilical cord blood-, and amniotic membrane-derived MSC could act as cell source for regenerative therapy to treat various disorders such as osteoporosis, arthritis, and vessel regeneration after injury [26–29]. MSC may also be induced to differentiate into functional neurons, corneal epithelial cells, and cardiomyocytes under specific pretreatments ex vivo and in vivo that broaden the capacity of these cells in regenerative therapeutic interventions [30–35]. In a previous study, umbilical cord matrix stem cells derived from human umbilical cord Wharton's Jelly were aimed to treat neurodegenerative disorders such as Parkinson's disease by transplantation into the brain of nonimmune-deficient, hemiparkinsonian rats [36]. Interestingly, phenotypic characterization of umbilical cord matrix-derived stem cells revealed a similar surface marker expression pattern to mesenchymal stem and progenitor cells (positive for CD10, CD13, CD29, CD44, and CD90 and negative for CD14, CD33, CD56, CD31, CD34, CD45, and HLA-DR). The transplantation resulted in a significant reduction of rotator behavior as a symptom for Parkinson's disease, thus suggesting an additional therapeutic role for umbilical cord matrix stem cells (MSC) in treating central nervous disorders [36].

These findings were enough evidences for scientists to speculate a promising role for MSC in regenerative therapy. In the past years, MSC have been used in clinical trials aiming for regeneration of tissues such as bone [37] and cartilage [38] as well as treatment of disorders such as spinal cord injury [39], multiple sclerosis (MS), Crohn's disease [2, 40], and graft-versus-host disease (GvHD) [41] due to their broad differentiation capacity and potential of hematopoietic cell recruitment [5, 42, 43].

Several clinical trials are running to identify different aspects of MSC application in terms of safety and efficacy.  Table 1 indicates a number of clinical trials using MSC for various treatments and regenerative interventions. As of date (07.10.2016), a total number of 657 clinical studies were found that involve mesenchymal stem cells for different clinical phases.

## 2. Stem Cells as Potential Tools for Regenerative Therapy: Promise and Perils 

In the recent decade, somatic stem cells have become attractive tools for cell therapy and regenerative medicine due to their proliferation and differentiation potential as well as established isolation and propagation protocols that promote a high standard of purity and functionality of the cells when applied in vivo. Adult stem cells (ASC) and progenitors, in particular mesenchymal stem cells have been derived from a variety of tissues such as umbilical cord and umbilical cord blood, placenta, bone marrow, epithelium, and white adipose tissue. These cells have been characterized, expanded, and applied for transplantation procedures in which allogeneic adult stem cells give rise to committed cells such as osteocytes, adipocytes, and chondrocytes as well as functional vessels in the process of neovasculogenesis [44–49].

The proliferation rate of adult stem cells and in particular MSC is a crucial parameter for stem cell therapeutic interventions like patient-specific tissue regeneration. There are, however, limitations with regard to the amount of tissue that can be taken from the patient, the limited propagation capacity of the cells that are isolated from the tissue, and restrictions in terms of passage number of the cells to be utilized for regenerative therapy. Therefore, it has been of great interest to enhance the proliferation rate of (mesenchymal) stem cells, especially in terms of patient-specific regenerative therapy. In this regard, low-level laser therapy (LLLT) has been tested in vitro to stimulate and enhance proliferation capacity of the cells. According to systematic review conducted by Ginani et al., LLLT is increasingly used as a method to enhance proliferative potential of adipose tissue-, dental pulp-, periodontal tissues-, and bone marrow-derived stem cells to date [50]. Ballini et al. showed that LLLT irradiation promotes proliferation capacity of dental pulp stromal cells and enhances the expression of proteins that are involved in osteogenesis [51].

Considering the tissue-specificity property of stem cells in determining their regenerative potential, it is of interest to test and compare the impact of LLLT on proliferative potential of stem cells that are derived from different tissues to ensure a more effective regenerative strategy approach.

MSC are, however, not considered as the only cellular mediators for enhancement of regenerative therapy, as embryonic stem cells (ESC) and, later on, induced pluripotent stem cell (iPSC) technology through cellular reprogramming were introduced and aimed to push regenerative cell therapy beyond its existing limits.

Pluripotent, inner blastocyst cell mass-derived cells are defined as embryonic stem cells (ESC) that can proliferate without limitation, possess the potential of self-renewal, and are able to differentiate into different cell types derived from all the three germ layers [52]. These characteristics together with the human embryonic stem cell (hESC) capability to differentiate into human adult cells led to the speculation that hESC might be useful for allogeneic cell transplantation research as well as clinical trials for treatment of diseases such as spinal cord injury, cardiovascular disorders, and diabetes [53, 54].

Differentiating hESC to numerous cell types including osteoblasts, cardiomyocytes (CM), hepatocytes, neurons, and endothelial cells (EC) to be used in cell replacement therapy (CRT) has been increasingly taken into consideration [55]. However, the procedure of deriving tissue-specific cells from hESC is challenging and requires establishment of reproducible methods for therapeutic interventions. A number of studies focusing on hESC differentiation into tissue-specific CM that do not express stemness markers are still in progress [56]. Moreover, CM populations derived from hESC have been shown to respond to drug stimuli and thus are suitable for assessment and development of small molecule therapeutics ex vivo [55, 57].

During the past few years, several studies were carried out to investigate differentiation of ESC into dopamine-producing neural cells [58, 59] and bone tissue [60] which can shed light to the future clinical trials using hESC to treat spinal cord injuries and bone damage.

ESC research offers great promise for understanding mechanisms of cell differentiation which ultimately leads to discovery of novel treatments for diseases such as myocardial infarction [61, 62]. Pluripotent stem cells can readily be cultured in vitro and can differentiate into all types of committed cells [61, 63]. With the ongoing progress in the field of ESC and regenerative medicine, these cells could be induced to differentiate into variety of committed cells that could be used for therapeutic interventions such as regenerative transplantation. Embryonic stem cells (ESC) were therefore identified as potential playmakers for regenerative therapy.

The therapeutic potential and benefit of ESC, however, have been a matter of debate and raised ethical concerns due to the opinion that the process of deriving embryonic stem cells results in severe damage to the embryo. Moreover, the existing complications and some as-of-yet unclarities in differentiation potential and proliferation rate of ESC pose risk of undesired complications such as teratoma formation and cancer development. Therefore it is not an approved procedure in several countries. Although research has overcome many of these limitations to date, ESC are still not fully approved for being used in cell therapy procedures and regenerative application [64].

Other groups of potential playmakers in regenerative therapy, induced pluripotent stem cells (iPSC), have come to the scene by Takahashi and Yamanaka who successfully produced induced pluripotent stem cells (iPSC) using mouse embryonic and adult fibroblast cells and introducing four transcription factors SOX2, OCT 3/4, KIF4, and c-myc to cells [65]. Later, they generated iPSC also from human somatic fibroblasts and established reprogramming strategies to convert differentiated human adult cells into a pluripotent state. Park et al. were able to generate iPSC from adult, neonatal, and fetal primary cells of human including skin fibroblasts [66]. Consequently, patient- and disease-specific stem cell generation methods were developed as crucial steps towards modern regenerative medicine and cell therapy [67]. For instance, Maehr et al. generated type 1 diabetes-specific iPSC from patients by reprogramming their fibroblasts with three transcription factors (OCT4, SOX2, and KLF4) with the potential of differentiating into insulin-producing cells that could be used to treat type 1 diabetes [68]. In 2012, John B. Gurdon and Shinya Yamanaka were jointly awarded the Nobel Prize in Physiology or Medicine for discovery of the path through which differentiated cells can be reprogrammed to become pluripotent.

Several studies on the implication and capacity of iPSC technology for therapeutic approaches have been carried out through which iPSC were generated from committed and somatic cells [69, 70]. These studies investigated their cellular, molecular, and functional properties and compared them with pluripotent and multipotent stromal cells. A differentiation protocol was investigated by Moslem et al. through which human iPSC derived-MSC were generated from fibroblasts and bone marrow-derived mesenchymal stem cells (BM-MSC) [70]. The iPSC-MSC generated in this study expressed a surface marker profile similar to that of normal BM-MSC, while having a shorter population doubling period, therefore possessing a more advanced proliferation capacity. Furthermore, iPSC-MSC revealed immunomodulatory properties through eliminating the proliferation capacity of CD4^+^ cells and reducing proinflammatory cytokines in a lymphocyte population admix [70].

Kang et al. also established a method for generating iPSC-MSC with morphological characteristics and surface marker expression profile similar to that of BM-MSC [69]. The iPSC-MSC generated in this study revealed osteogenic and chondrogenic differentiation capacity comparable to those of BM-MSC, but they revealed less efficiency in terms of adipogenic differentiation capacity [69].  Table 2 indicates a selection of genes and primers that have been involved in human iPSC-MSC technology studies [69–73].

The iPSC technology has undoubtedly raised hope in regenerative biology; however its use in regenerative medicine did not appear as a facile, straight-forward procedure, as iPSC technology led to complications in cell therapy and regeneration [74]. The genetic stability in reprogrammed cells has not been proven to remain constant [75, 76] and because of genetic alterations, these cells have not been considered as reliable tools for clinical use in transplantation and regeneration to date.

In comparison with ESC and iPSC, adult somatic stem cells do not cause ethical and severe health issues and are therefore widely used in regenerative research. There have been, however, limitations concerning the in vitro expansion and pluripotency when using adult somatic stem cells for therapy [64]. Nevertheless, lower risk in terms of application, low incidence of post-therapy complications, and less ethical concerns compensate the limitations of ASC in terms of expansion rate and pluripotency to a significant extent.

Further investigations are still required for application of ESC and iPSC in regenerative medicine until these cells would be considered as effective tools for clinical regenerative therapy. For this reason, other options such as new sources of ASC, in particular, MSC as an important adult stem cell subfamily, have been considered for establishment of successful and progressive cell-based regenerative and therapeutic procedures. The need for novel cell sources is obvious because of increasing need of regenerative cell therapy for diseases that are, as of date, difficult, if not impossible to be cured.

## 3. Tissue Specific MSC: Diversity in Regenerative Potential 

MSC are present in several adult tissues. Despite similar morphology and phenotypic properties amongst MSC that have been isolated from various tissue sources, their regenerative potential has been shown to differ. It has been previously described that activated aging mechanism in MSC has an impact on their regenerative potential, probably due to DNA damage accumulation [77] and/or impairment of metabolic system as a result of mitochondrial damage [77, 78]. Nonetheless, several studies have been carried out that show differences in regenerative capacity of MSC populations of the same passage number that have been isolated from different sites [79–81]. The variability in regenerative potential of MSC populations that are derived from various tissues might be due to the impact of stem cell niche on cell fate, known as stem cell niche theory [82], genetic variability, and/or epigenetic alterations.

### 3.1. Bone Marrow-Derived MSC

Bone marrow (BM) was one of the first tissues that had been used for isolation and propagation of mesenchymal stem and progenitors. Bone marrow aspirate is rich in hematopoietic and nonhematopoietic stem cells, endothelial progenitor cells (EPC) derived from embryonic hemangioblasts, and mesenchymal stem cells (MSC). MSC have been shown to participate in hematopoiesis or bone marrow regeneration [83, 84]. They also have the potential to give rise to “pericytes,” the perivascular cells on the outer layer of vessels supporting the stability of capillaries and directing the blood flow [23]. Human BM-MSC have been shown to successfully participate in neovasculogenesis and collaborate with endothelial colony forming cells for establishment of perfused microvessels in vivo [85–87]. BM-MSC have been considered as gold standard tools for osteogenic and chondrogenic regeneration. There have been, however, increasing reports on the role of other source-specific MSC such as umbilical cord blood- (UCB-) MSC and adipose tissue- (AT-) MSC in promoting osteogenic and chondrogenic differentiation in vitro and in vivo [88, 89].

### 3.2. Adipose Tissue-Derived MSC

Adult adipose tissue is rich in fibroblast-like cells with multidifferentiation potential [90–92]. In 2001, these cells were identified as MSC [93], leading the adipose tissue (AT) to be recognized as a source of MSC isolation. Reports on the regenerative potential of AT-MSC showed that they are potent in contributing to vessel formation [94] and act as pericytes as well as being able to differentiate into bone (osteoblasts) [89, 95] and cartilage (chondrocytes) [96, 97]. These cells were isolated from the liposuction material and they expressed potential to undergo osteogenic, adipogenic, myogenic, and chondrogenic differentiation in vitro [98]. It has been shown that AT-MSC have the potential to differentiate into hepatocyte-like cells in the presence of certain growth factors such as hepatocyte growth factor (HGF) and fibroblast growth factors 1 and 4 (FGF1, FGF4) [99–101]. These hepatocyte-like cells have been shown to express phenotypes such as albumin secretion and lipoprotein absorbance that are known as liver-specific markers. Moreover, these cells have been shown to home into the liver parenchyma after being transplanted into the liver [99]. Reports show a broad range of regenerative potential attributed to the AT-MSC, from soft tissue regeneration (hepatocyte regeneration and vasculogenesis) to hard tissue formation (osteogenesis).

### 3.3. Umbilical Cord and Cord Blood as MSC Sources

Several studies revealed that cells isolated from Wharton's Jelly (WJ), a component of umbilical cord extracellular matrix, express stemness characteristic and multipotency [102, 103]. These cells also express biomarkers similar to those of bone marrow mesenchymal stem cells (BM-MSC). Mesenchymal stem cells derived from Wharton's Jelly within the umbilical cord have been shown to give rise to various cellular types of nerve system and connective tissue [104, 105]. Umbilical cord-derived mesenchymal stem cells (UC-MSC) express biomarkers such as Nanog and Oct3/4A [104]. These cells have been known as hypoimmunogenic cells due to their ability to modulate NK cells and promote regulatory T-cell expansion [104, 106, 107].

The potential of UC-MSC to participate in neovasculogenesis [85, 108, 109] and differentiate into hepatocyte-like cells [110] strongly suggests that UC-MSC can give rise to various cell types, which indicates the ability of UC-MSC to go beyond lineage borders. Considering their proliferation potential in vitro and their immunoregulatory properties, these cells are extremely promising for regenerative applications in various treatment settings [106].

There have been, however, contradictory reports in terms of surface markers that are expressed on UC-MSC surface [111]. According to ISCT report, CD105 is a required surface marker for verification of MSC [5]. However, several reports contradict each other, as in some studies CD105 has been shown to be present on UC-MSC surface [112–114] and its expression is constant even in different, long-term cell passages [115], whereas a number of reports have argued against the ability of UC-MSC to express CD105 as a surface marker. These studies claim that even though CD105 is expressed in UC-MSC, the expression of this surface marker is detectable up till passage 5 [116, 117].

UC-MSC have been shown to maintain a high differentiation potential in vitro as these cells have shown the ability to differentiate into adipocytes, chondrocytes, osteoblasts, muscle cells, cardiomyocytes, beta cells, endothelial cells, neurons and dopaminergic neurons, and so forth [111, 118–121].

It has been shown that regenerative potential of UC-MSC can differ if the cells are obtained from an individual with metabolic disorders such as type 1 diabetes. Kim et al. indicated that UC-MSC derived from diabetic pregnant women show lower potential of osteogenic and adipogenic differentiation, whereas their surface marker expression profile is not significantly affected. The cell population doubling has also been shown to diminish in UC-MSC from diabetic mothers when compared to UC-MSC from healthy individuals [122]. This finding leads us to conclude that metabolic disorders of the mother have an impact on biological properties of UC-MSC, which attributes to the baby. This has to be taken into consideration when choosing a cell source for clinical application and/or in case of patient-specific clinical regenerative strategies.

Umbilical cord blood (UCB) has always been considered as a source of hematopoietic stem cells (HSC) [10, 123]. Nonetheless, recent findings suggest that UCB serves as a source of MSC with a high regenerative potential [10]. It has been revealed that UCB-MSC can differentiate into osteoblasts, chondrocytes, and pericytes in course of vessel formation [85, 86, 124]. The phenotypic characterization of UCB-MSC has been shown to be consistent with that of BM-MSC [125]. There have been reports on UCB-MSC ability to differentiate into neuron-like cells [126] under certain conditions, which indicate their ability to give rise to cells of all three germ layers [124, 126].

### 3.4. Dental Tissue-Derived MSC

Dental tissues are specialized tissues and they do not undergo continuous remodeling as has been indicated in other bony tissues; therefore, stem cells that are obtained from dental tissue might show a restricted differentiation capacity compared to BM-MSC [127, 128].

Dental pulp stem cells (DPSC) are amongst different human dental stem and progenitor cells that have been isolated and characterized to date [128]. DPSC possess self-renewal and differentiation capacity. Human pulp cells can differentiate into odontoblastic cells in vitro, possessing polarized cell bodies and the ability to accumulate mineralized nodules [129–131]. Although dental tissue-derived stem cells are obtained from specialized tissue and they are most potent for differentiation into odontogenic cells, DPSC also have the potential to differentiate into other cells such as adipocytes and neurons [132]. Recently, it has been revealed that DPSC have the potential to give rise to chondrocytes, osteoblasts, and myocytes in vitro [133, 134]. To date, the regenerative application of dental pulp-MSC involves regeneration of the whole tooth and partial bony substrate of the oral cavity in the process of maxillofacial surgical interventions [135–137].

The osteogenic differentiation potential of the cells isolated from dental follicle (DF) has been investigated by Mori et al. [138]. This study has revealed that stemness markers are released by dental bud stem cells. Upon differentiation, these cells have been shown to express osteoblastic biomarkers such as collagen I and alkaline phosphatase (ALP) which indicates their commitment to osteoblast-like lineage [138]. Moreover, a recent report involving the role of integrin and cadherin in differentiation of dental bud stem cells has unraveled a crucial role for integrin *α*V*β*3 during differentiation of these stem cells into osteoblasts [139]. The data elucidates the impact of extracellular matrix (ECM) proteins in directing stem cell fate towards bone formation [139].

The studies that have been carried out on dental stem cells and their regenerative potential have raised promise for using dental tissue-derived MSC in fracture healing as well as regenerative bone formation interventions due to disease or loss of the tissue [137].

### 3.5. Amniotic Membrane-Derived MSC

The amniotic membrane is a part of the placenta that protects the fetus during pregnancy and provides nutrient transport to fetus [140]. The amniotic membrane is known as an efficient scaffold for treatment of burns as well as during skin and corneal transplantation, since this tissue possesses anti-inflammatory property [141]. To date, the amniotic membrane is widely used as a material for clinical interventions. Decellularized amniotic membrane can serve as a scaffold and can be used for transplantation interventions.

Amniotic membrane-derived mesenchymal stem cells (AMN-MSC) have been shown to have the potential to differentiate into all three mesodermal lineage cells as well as endodermal lineage cells [142]. They have been shown to express mesenchymal surface markers such as CD105 and CD90 while lacking the hematopoietic markers such as CD29, CD34, and CD45 [143]. Moreover, it has been revealed that the amniotic membrane of placenta can express antiangiogenic and anti-inflammatory components [144]. These results further justify the potential of AMN-MSC application in regenerative medicine, since overcoming inflammation and immunogenicity issues is amongst the most important challenges for a successful outcome of regenerative transplantation. Interestingly, despite expression of pluripotent markers like Oct-4, Nanog, TRA-1-60 and TRA-1-81, AMN-MSC do not cause teratoma formation [145]. An intact amniotic membrane (AMN) promotes secretion of anti-inflammatory and antifibrosis components. It also lacks vasculature structures as well as neurons, which makes AMN a suitable scaffold for wound healing [146, 147].

## 4. Tissue Specific Regenerative Potential of MSC 

The regenerative potential of MSC isolated from different tissues has been shown to undergo alteration according to the tissue of isolation [148, 149]. It has been shown that BM-MSC possess a higher potential in giving rise to osteoblasts and chondrocytes [79, 149], whereas adipose tissue-derived MSC (AT-MSC) have been shown to contribute more successfully to capillary-like network formation in vitro [150] as well as vasculogenesis in vivo [85, 86]. Umbilical cord blood- (UCB-) MSC also showed a high potency in giving rise to pericytes during vasculogenesis [86], whereas their potential for osteogenic differentiation has been shown to diminish compared to BM-MSC [151], which still play as the gold standard for osteogenic differentiation and regeneration.

AMN-MSC were also shown to successfully participate in neurogenesis, whereas such a regenerative potential has not been distinguished in UC-MSC [152, 153]. Amniotic membrane-derived MSC, however, have not been shown to participate in the process of vasculogenesis as successfully as UC-, UCB-, AT-, and BM-MSC did [86].

Despite the fact that DPSC and BM-MSC are regulated by similar factors and they also possess a similar protein expression profile, these populations have been shown to alter significantly in their proliferative capacity in vitro and, more importantly, in their regenerative capacity in vivo [154]. BM-MSC give rise to bone tissue in the mouse model under treatment as described in studies [155, 156]. The chondrogenic and adipogenic potential of BM-MSC has been higher compared to that of DPSC [157, 158]. Conversely, the neurogenic differentiation potential of dental mesenchymal stem cells might be more robust compared to that of BM-MSC, since these cells possess neural crest origin [127].

BM-, dental pulp- (DP-), and adipose tissue- (AT-) derived MSC have revealed a greater promise in regenerative therapy since these adult stem cells might promote patient-specific regenerative interventions.

## 5. MSC in Regenerative Therapy 

MSC are attractive alternatives for regeneration of the injured and/or deficient cells and tissues due to their multipotent differentiation capacity as well as their immunomodulatory and anti-inflammatory properties through cellular crosstalk and production of bioactive molecules [159]. MSC have the unique potential either to directly participate in regeneration and repair processes or to play an immune modulatory role to enhance treatment of autoimmune diseases such as type 1 diabetes (T1D).

### 5.1. The Role of MSC in Neovasculogenesis

The combination of multipotent endothelial progenitor cells (EPC) and mesenchymal stem cells (MSC) is an additional key tool for stem cell therapy. These cells are localized in bone marrow stroma as well as vascular inner and outer layer and perivascular niches and are capable of forming mature endothelial cells and mesenchymal cell lineages such as osteoblasts, chondrocytes, adipocytes, and myoblasts [30, 83, 160]. EPC derived from bone marrow, inner vascular wall, umbilical cord, and umbilical cord blood as well as circulating EPC are of great importance for clinical trials and cell therapy procedures. Being capable of migrating through the circulation and differentiating into committed endothelial cells, EPC are crucial mediators for promoting angiogenesis and de novo vasculogenesis as well as endothelium repair in case of vascular damage [25, 84, 161, 162]. It has been previously revealed that SDF-1 can be expressed by activated thrombocytes within blood flow, which is responsible for EPC recruitment to artery structures in vivo [163]. This shows the potential of EPC to participate in vascular repair of damaged peripheral tissues. As has been indicated in a previous study, isolation and transplantation of a human EPC subpopulation (negative for CD34 and CD14, positive for CD133 and VEGFR2) in nude mice with damaged artery resulted in a repaired endothelial layer and wound healing caused by the injected EPC subpopulation [161]. In addition, bone marrow-derived MSC within perivascular niche have been shown to form bone marrow stroma, bone, cartilage, adipose tissue, and myocytes in vivo [26, 30, 84]. Early signaling signature during stem cell mediated vessel formation has been investigated by Rohban et al. [85]. In this study, a coculture approach of mesenchymal stem cells and endothelial colony forming cells revealed that the two progenitor cells collaborate to form stable and perfused microvessels. Moreover, the study revealed that MSC and endothelial progenitor cells communicate through signaling molecules and pathways such as caspase and mitogen activated protein kinase (MAPK) to direct their fate toward vessel formation.

**Figure 1:**
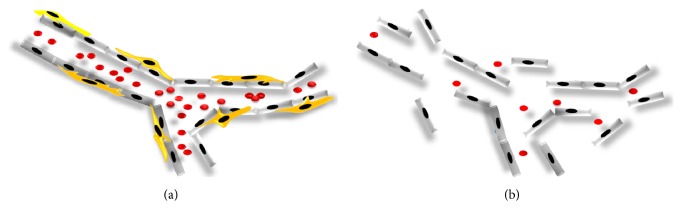
(a) MSC and ECFC collaborate to form stable, perfused, and functional vessels in vivo. The inner layer of the vessel is established by ECFC (grey), whereas MSC (yellow) form the outer layer of neovessel to support the stability and functionality of the vasculature. (b) Unstable vessel. In the absence of mesenchymal stem cells, the inner layer of the neovessel raptures due to the lack of pericytes which play a crucial role for maintenance of vasculature stability in vivo.

Mitogen activated protein kinases (MAP kinases) have been also shown to regulate MSC differentiation to osteo- or adipogenic lineage [164] with a significant expression of p38, Erk2, and JNK2 in a time-dependent manner [164] suggesting a crucial role for protein kinase signaling molecules and their phosphorylation status during differentiation. The coculture of MSC and ECFC has been shown to result in vascular structure formation in vivo. The vessels have been shown to remain stable and functional up to 6 months after transplantation [85]. This finding justifies the supportive role of MSC for maintaining the stability and functionality of neovessels.  Figure 1(a) depicts the contribution of MSC and endothelial colony forming cells (ECFC) in the process of neovasculogenesis.  Figure 1(b) shows the formation of neovessels in the absence of MSC resulting in the formation of unstable vasculature.

### 5.2. The Role of MSC in Osteogenesis and Chondrogenesis

Bone and cartilage injuries occur as a result of bone fracture, or joint diseases such as rheumatoid arthritis or osteoarthritis. These disorders have a costly economic and social impact on the quality of life amongst middle-aged patients. Despite the progress in orthopedic surgery, bone and cartilage repair have remained a major challenge because large injuries do not heal spontaneously [165–169]. The regeneration of ruptured/injured cartilage in a variety of diseases such as degenerative osteoarthritis and herniation is a major goal in cartilage regeneration studies [167, 168, 170, 171].

Studies on mesenchymal stem cells have opened a new horizon for bone and cartilage tissue engineering. Because of their multipotent capacity, MSC lineages have been successfully used in animal models to repair articular cartilage and regenerate bone [35, 165, 170, 172, 173]. Recent research studies have indicated that bone and cartilage might be repaired through percutaneous implantation of MSC [170, 172–175].

The potential of MSC and progenitor cells in prospective cell-based regenerative models has been investigated by Lohberger et al. [176]. The study investigated MSC isolated from three different intraoral bone sites, as well as dental pulp with regard to their potential of differentiating into osteogenic, adipogenic, and chondrogenic lineages. It has been shown that mesenchymal stromal cells isolated from these sites have the potential of osteogenic, but also adipogenic and chondrogenic differentiation in vitro [176].

Human mesenchymal stromal cells isolated from bone marrow (BM) and alveolar bone have been compared according to their regenerative potential by Pekovits et al. [177]. The study aimed to evaluate the potential of bone marrow (BM) and alveolar-derived MSC for regenerative applications in maxillofacial and oral tissue engineering. The results showed multilineage differentiation potential (osteogenic and chondrogenic differentiation) of alveolar bone-mesenchymal stem cells in vitro, which was comparable to that of BM-MSC in vitro [177].

Complete healing occurs when the regenerated tissue has been integrated into the neighboring host tissue and the differentiation process has been thoroughly performed [178, 179]. However, complete bone and cartilage healing is still highly demanding and complete differentiation into functional cartilage has not yet been achieved. Complete healing might be achieved by establishing novel strategies for using scaffolds in combination with pretreated and/or untreated MSC in the presence of selective differentiation factors [178, 180–182]. The long-term behavior of MSC in combination with growth factors and bioscaffolds implanted in morbid joints remains to be studied prior to any clinical application in disorders such as osteoarthritis or rheumatoid arthritis [182–184].

### 5.3. MSC as Tools for Cornea Regeneration

As indicated earlier, MSC can differentiate into different mesodermal cells and they also possess transdifferentiation ability to preserve phenotypes of neural ectodermal and epithelial cells [185]. It has been shown that BM-MSC can mimic limbal fibroblast cells which are crucial in maintenance of epithelial stem cells in the limbal niche [186]. Both BM-MSC and limbal fibroblasts have been shown to express a similar surface marker profile, including CD106, CD54, CD166, CD90, CD29, CD71, and CD105. Moreover, both BM-MSC and keratocyte cell types express CD13, CD29, CD44, CD56, CD73, CD90, CD105, and CD133 biomarkers and lack HLA-DR, CD34, CD117, and CD45 on their surface [187]. These studies suggest that MSC can be induced to differentiate into corneal cells. However, there is no in vivo evidence which indicates differentiation of MSC to corneal epithelial cell types. Nevertheless, in vitro differentiated cells can be used in corneal tissue regeneration or treatments that involve tissue/cell replacement.

During development, surface ectoderm gives rise to the corneal epithelium [188]. It has been hypothesized that MSC might be reprogrammed to ectodermal lineage cells. A study conducted by Ma et al. indicated that the MSC population that was transplanted to cornea failed to differentiate into epithelial cells in vivo [32]. In this study, human BM-MSC were applied on amniotic membrane, serving as scaffold, and transplanted on the chemically injured rat cornea. The study revealed that BM-MSC can survive and cause cornea inflammation but did not undergo corneal epithelium differentiation [32].

In a preclinical study using rabbits, BrdU labelled BM-MSC were seeded on fibrin scaffolds and were transplanted into the alkali damaged cornea. The BrdU positive cells were shown to participate in the process of cornea healing which clearly indicated the ability of BM-MSC to differentiate into corneal epithelial cells [189].

The result of several in vitro experiments supported the idea that MSC are able to resemble cornea epithelial cell phenotype under certain conditions; however, up till now, the in vivo data has not shown supportive evidence that justifies the in vitro results.

Recently, adipose tissue-derived MSC (AT-MSC) have shown the ability to differentiate into the corneal epithelium [190]. Although several scientific groups have reported the differentiation of MSC into corneal epithelial cells, the precise mechanism remains unclear and deserves further investigation.

A number of studies have revealed the potential of umbilical cord mesenchymal stem cells (UC-MSC) and bone marrow mesenchymal stem cells (BM-MSC) to differentiate into corneal endothelial cells [191, 192]. However, the characteristics and functions of endothelial cells have not been precisely studied and need to be further investigated.

### 5.4. Immune Modulatory and Regenerative Potential of MSC in T1D

Immune-mediated disorders like type 1 diabetes (T1D) severely affect quality of life in several millions of patients all over the world. T1D leads to a shorter life span of the patient, has various side effects including cardiovascular and ophthalmic disorders and neuropathy. The disease puts economic pressure both on the health system and the patient. Therefore, great effort has been made to develop innovative therapeutic strategies for cell-based therapy through stem cell immune modulation, autologous/allogeneic stem cell transplantation, and small molecule mediated beta cell regeneration for treatment of T1D.

The use of MSC in cell-based therapy in T1D has been investigated by a number of scientific groups all over the world [193–203].

The potential of bone marrow-derived MSC in immunomodulation of immune-mediated disease T1D and cell-based regenerative models has been investigated by Fiorina et al., 2009 [197]. In this study, murine MSC isolated from bone marrow (BM) have been characterized with regard to their potential to modulate immunity in T1D. The results have revealed that transplantation of stromal cells from BALB/C mice but not from NOD mice into mice that were prone to diabetes delayed the onset of diabetes development. This data suggests that allogenic transplantation of MSC from a healthy donor leads to a better therapeutic outcome compared to autologous transplantation in diabetic mice. The study also showed that mouse-derived mesenchymal stromal cells isolated from BM have the potential of osteogenic, adipogenic, and chondrogenic differentiation in vitro [197].

Human mesenchymal stromal cells isolated from BM and peripheral blood (PB) have been tested in a humanized mouse model by Lee et al., 2006 [204]. In this study immune-deficient mice that have been rendered diabetic by means of streptozotocin (STZ) were used to study the impact of human MSC in treatment of diabetes. This study showed that infusion of human MSC eliminates glucose levels and increases insulin levels in peripheral blood. Human DNA was also detected in mice kidney and pancreas which elucidates homing of human MSC in those tissues persumably for immunomodulatory/regenerative purposes.

Other studies have also focused on MSC derived from adipose tissue (AT) [201] and placenta [205]. According to the study, AT-MSC play a protective role for beta cells in diabetic animal models [201]. Talwadekar et al. have also compared immunomodulatory properties of placenta-derived MSC to those of cord-derived stromal cells [205] suggesting enhanced immunomodulatory properties for placenta-derived MSC compared to cells that are isolated from other birth-derived tissues, for instance, umbilical cord. The regeneration of insulin-producing beta cells and the use of immunomodulatory effect of stem cells in a variety of autoimmune and/or immune-mediated diseases like T1D are major goals in relevant clinical studies nowadays.

Investigations on the mesenchymal stromal cells have opened a new horizon for diabetes research. Because of their multipotent capacity, MSC lineages have been used successfully in animal models to suppress immune reactions that cause beta cell death and the onset of T1D [206]. Recent research studies have indicated that beta cells within pancreatic islets might be repaired through transplantation/infusion of MSC [194, 195, 207–209]. Other studies also showed that MSC transplantation in animals or patients with T1D can reverse the disease [195, 208]. However, most of studies showed that allogenic transplantation is more efficient in reversing diabetes rather than autologous transplantation [197, 198, 204].

## 6. Conclusion 

Stem cells derived from a variety of sources are promising tools for regenerative cell therapy. Although stem cell therapy has opened a new horizon in regenerative medicine, there are still several obstacles that need to be overcome before this novel treatment tool can be used in large scale in clinics. However, it is obvious that regenerative stem cell therapy has been transformed from scientific fiction to a feasible medical procedure. Regenerative stem cell therapy has created a lot of hope amongst scientists and physicians for finding more effective treatment strategies; nevertheless, it is essential for this new spectrum to develop further through high quality investigations and an effective contribution of researchers and physicians to perform advanced clinical trials aiming to facilitate MSC application for clinical therapy.

## Figures and Tables

**Table 1:** A selection of registered clinical trials on the basis of MSC as the relevant therapeutic tool (https://www.clinicaltrials.gov).

	Title	Recruitment	Conditions	Phases	Intervention	Sponsors
1	Mesenchymal Stem Cells in Knee Cartilage Injuries	Completed	Articular cartilage disorder of knee	Phase II	Biological: autologous mesenchymal stem cells	University of Jordan
2	“One-Step” Bone Marrow Mononuclear Cell Transplantation in Talar Osteochondral Lesions	Recruiting	Osteochondritis	Phase III	Procedure: bone marrow cells transplantation on collagen scaffold	Istituto Ortopedico Rizzoli
3	Mesenchymal Stem Cell Based Therapy for the Treatment of Osteogenesis Imperfecta	Active, not recruiting	Osteogenesis imperfecta	Phase I	Biological: mesenchymal stem cells	Hospital de Cruces; Hospital Universitario de Getafe; Hospital Infantil Universitario Niño Jesús, Madrid, Spain
4	Treatment of Patients With Newly Onset of Type 1 Diabetes With Mesenchymal Stem Cells	Completed	Type 1 diabetes mellitus	—	Biological: mesenchymal stem cells	Uppsala University Hospital
5	Mesenchymal Stem Cells for Multiple Sclerosis	Recruiting	Multiple sclerosis	Phase I Phase II	Drug: mesenchymal stem cells; drug: suspension media	University Hospital, Toulouse
6	Autologous Mesenchymal Stem Cells Transplantation in Cervical Chronic and Complete Spinal Cord Injury	Recruiting	Spinal cord injury	Phase I	Biological: autologous mesenchymal cells transplantation	Hospital Sao Rafael

**Table 2:** Selected genes and primers involved in human iPSC-MSC technology studies.

Gene	Implication	Primer sequence 5′-3′ Forward
Human peroxisome proliferator-activated receptor *γ* (PPAR*γ*)	Proliferation capacity	CTAAAGAGCCTGCGAAAG
Human peroxisome proliferator-activated receptor *α* (PPAR*α*)	Proliferation capacity	ACTCCGTCTTCTTGATGAT
Octamer-binding transcription factor 4 (OCT4)	Stemness	CCTCACTTCACTGCACTGTA
Kruppel-like factor 4 (KLF4)	Stemness	GATGAACTGACCAGGCACTA
Myc (C-MYC)	Stemness	TGCCTCAAATTGGACTTTGG
Sex determining region Y-box 2 (SOX2)	Stemness	CCCAGCAGACTTCACATGT
Lin-28 homolog A (LIN28)	Stemness	AGTAAGCTGCACATGGAAGG
Collagen 2 (COL2a)	Chondrogenesis Osteogenesis	TCTACCCCAATCCAGCAAAC
Runt-related transcription factor 2 (RUNX2)	Osteogenesis	CAGTAGATGGACCTCGGGAA
Aggrecan (ACAN)	Chondrogenesis	CTGGACAAGTGCTATGCCG
Alkaline phosphatase (ALP)	Chondrogenesis Osteogenesis	CAACAGGGTAGATTTCTCTTGG
Osteocalcin (OC)	Osteogenesis	AGTCCAGCAAAGGTGCAGCC
